# Elucidation of the Structure of Lignin–Carbohydrate Complexes in Ginkgo CW-DHP by ^13^C-^2^H Dual Isotope Tracer

**DOI:** 10.3390/molecules26195740

**Published:** 2021-09-22

**Authors:** Kai Zhang, Yanchao Liu, Sheng Cui, Yimin Xie

**Affiliations:** 1 Research Institute of Pulp & Paper Engineering, Hubei University of Technology, Wuhan 430068, China; zk827967161@163.com (K.Z.); lyc10051813@163.com (Y.L.); Cui2019hbut@163.com (S.C.); 2 Hubei Provincial Key Laboratory of Green Materials for Light Industry, Hubei University of Technology, Wuhan 430068, China

**Keywords:** lignin–carbohydrate complexes, CW-DHP, isotope labelling, ginkgo, NMR

## Abstract

To elucidate the chemical linkages between lignin and carbohydrates in ginkgo cell walls, ^13^C-^2^H-enriched cell wall-dehydrogenation polymers (CW-DHP) were selectively prepared with cambial tissue from *Ginkgo biloba* L. by feeding D-glucose-[6-^2^H_2_], coniferin-[α-^13^C], and phenylalanine ammonia-lyase (PAL) inhibitor. The abundant detection of ^13^C and ^2^H confirmed that D-glucose-[6-^2^H_2_] and coniferin-[α-^13^C] were involved in the normal metabolism of ginkgo cambial cells that had been effectively labelled with dual isotopes. In the ginkgo CW-DHP, ketal and ether linkages were formed between the C-α of lignin side chains and carbohydrates, as revealed by solid state CP/MAS ^13^C-NMR differential spectroscopy. Furthermore, the DMSO/TBAH ionic liquids system was used to fractionate the ball-milled CW-DHP into three lignin-carbohydrate complex (LCC) fractions: glucan–lignin complex (GL), glucomannan–lignin complex (GML), and xylan–lignin complex (XL). The XRD determination indicated that the cellulose type I of the GL was converted into cellulose type II during the separation process. The molecular weight was in the order of Ac-GL > Ac-GML > XL. The ^13^C-NMR and ^1^H-NMR differential spectroscopy of ^13^C-^2^H-enriched GL fraction indicated that lignin was linked with cellulose C-6 by benzyl ether linkages. It was also found that there were benzyl ether linkages between the lignin side chain C-α and glucomannan C-6 in the ^13^C-^2^H-enriched GML fraction. The formation of ketal linkages between the C-α of lignin and xylan was confirmed in the ^13^C-^2^H-enriched XL fraction.

## 1. Introduction

Lignocellulosic biomass has attracted widespread attention as one of the most important raw materials for energy, chemicals, and materials because of its unique sustainability and renewability. Lignocellulose is made up of three types of biopolymers: cellulose, hemicelluloses, and lignin. These components are cross-linked in various covalent and non-covalent ways to form a tightly bonded network. The lignin–carbohydrate complexes (LCC) [1,2] were formed by covalently linking hydrophobic lignin with hydrophilic polysaccharides. The natural LCC found in plant cell walls and the LCC formed during the acquired processing seriously hinder delignification during chemical pulping [3,4,5]. When converting lignocellulose into bioethanol, the presence of the LCC structure restricts the enzymatic hydrolysis [6,7,8] and fermentation processes [9,10].

Three types of lignin–carbohydrate bonding have been proposed in the literature: benzyl ether, ester and phenyl glycosidic linkages [11,12,13]. The ether linkage was formed between the quinone methide intermediate of lignin and the alcoholic hydroxyl group of the sugar residue, whereas the ester linkage was formed between the quinone methide intermediate of lignin and the carboxylic group of uronic acid [11,14]. Traditional analysis methods, such as oxidation [15], acid hydrolysis [16] and alkali-catalysed hydrolysis [17] can also provide useful information about the LCC structure, but they may cause damage to the structure of lignin or polysaccharides and are ineffective in elucidating the structural characteristics of LCC. Therefore, the non-destructive and complete separation of the lignin and carbohydrate bonding structures is a prerequisite for the study of the LCC structure. Some researchers [18,19,20] have suggested a fractionation method in which ball milling is combined with dimethyl sulfoxide (DMSO)/tetrabutylammonium hydroxide (TBAH) ionic liquids to completely dissolve LCC and separate different LCC fractions. Even though researchers have proven the existence of a covalent bond between lignin and carbohydrates, further research into the form and quantification of the covalent bond is still needed.

LCC is a macromolecular polymer with a small content of LC bonds; moreover, because of the low natural abundance of ^13^C isotope (about 1.1%), there is an extensive overlapping of carbohydrate and lignin signals in carbon-^13^ nuclear magnetic resonance (^13^C-NMR). The combination of ^13^C isotope tracing with ^13^C-NMR determination is a non-destructive analytical approach that can solve the issues mentioned above. Important information on the polymerisation process and the chemical cross-linking of cell wall polysaccharides and lignin may be properly obtained using this type of analysis. The ^13^C isotope labelling technique has been successfully used to study the chemical structure of LCC in ginkgo (*Ginkgo biloba* L.), oleander (*Nerium oleander*), rice stalk (*Oryza sativa*) and wheat straw (*Triticum aestivum*) [11,21,22,23,24,25,26]. Through the preceding research, the existence of benzyl ether, benzyl ester, and ketal linkages between lignin and carbohydrates have been confirmed. However, the work mentioned above can only find information on linkages from one side of the LCC structure, such as polysaccharide structures or lignin structures, while the information about the other side, without labelling, relies only on speculation and chemical analysis. Moreover, the one-sided isotope labelling can hardly obtain quantitative information about the relationship between lignin and polysaccharides.

In this study, D-glucose-[6-^2^H_2_] and coniferin-[α-^13^C] dual isotopic tracer methods were applied to trace glucose and lignin, respectively. The selective ^13^C-^2^H-enriched cell wall-dehydrogenation polymers (CW-DHP) were prepared from ginkgo soft cambial tissues and fractionated to acquire three different LCC fractions, which were systematically characterised to evaluate the complex chemical bonds between lignin and carbohydrates.

## 2. Results and Discussion

### 2.1. Preparation of Ginkgo CW-DHP and Lignin–Carbohydrate Complex Fractions

**Figure 1:**
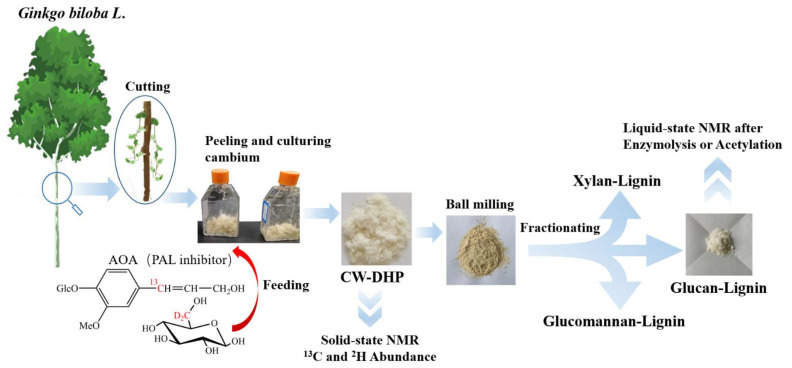
Schematic illustration of the preparation and analysis of specifically ^13^C-^2^H-enriched LCC fractions in ginkgo CW-DHP.

The preparation and analysis of the specifically ^13^C-^2^H-enriched lignin–carbohydrate complex fractions in ginkgo CW-DHP are schematically illustrated in Figure 1. The original ^13^C-^2^H double stable isotope technique was applied to trace lignin and carbohydrates via coniferin-[α-^13^C] and D-glucose-[6-^2^H_2_], respectively.

First, the selectively ^13^C-^2^H-enriched CW-DHP was prepared with cambial tissue from *Ginkgo biloba* L. β-glucosidase can promote the hydrolysis of coniferin-[α-^13^C] into coniferyl alcohol monomers and glucose units. Glucose can generate hydrogen peroxide in situ by glucose oxidase in cambial cells. Hydrogen peroxide combined with the phenol oxidase of wood cells can polymerize coniferyl alcohol monomers into CW-DHP. Wood cambial tissues naturally contain the enzymes necessary for the polymerization of coniferin into CW-DHP [27]. The ginkgo CW-DHP was then dissolved in ionic solutions and fractionated into three different LCC fractions. Finally, ^13^C-NMR and ^1^H-NMR were used to characterise the covalent bonds of LCC from the two perspectives of lignin and carbohydrates, respectively.

### 2.2. Characterisation of Gingko CW-DHP

#### 2.2.1. Abundance Characterization of ^13^C-^2^H-Enriched Ginkgo CW-DHP

According to Table 1, the δ^13^C (VPDB) and δD (VSMOW) of the experimental group, B, were much larger than those of the control group, A. The ^13^Cα/^12^Cα ratio of the ^13^C-^2^H-enriched ginkgo CW-DHP was 6.6 times that of group A, and the D6/H6 ratio of group B was 37 times that of the unenriched ginkgo CW-DHP. These two sets of data indicate that exogenous coniferin-[α-^13^C] and D-glucose-[6-^2^H_2_] are involved in the normal metabolism of ginkgo cambial cells, and that the lignin and polysaccharides of ginkgo CW-DHP were successfully labeled by ^13^C and D, respectively, after cultivation. These results were consistent with previous studies [11,26,28,29], and ginkgo CW-DHP could be used in subsequent experiments.

#### 2.2.2. Evaluation of the Lignification of Ginkgo CW-DHP

The total lignin content of the natural *Ginkgo biloba* L. xylem sample was measured to be 31.03% (±0.15%). The lignin content of the ginkgo soft cambial tissues determined by the acetyl bromide method before culture was 14.47% (±0.12%), which indicated that the degree of lignification was low and that the differentiation ability was strong, as shown in Table 2. After biological culture in the laboratory, the lignin content of the unenriched sample and the isotope-enriched sample increased by 19.74% (±0.16%) and 19.89% (±0.14%), respectively. This showed that when coniferin-[α-^13^C], D-glucose-[6-^2^H_2_] and AOA were administered, the ginkgo cambial tissues could be metabolised normally and differentiated into lignin and polysaccharides during 18 days of culture. Therefore, the lignification degree of ginkgo CW-DHP improved significantly. Furthermore, the lignin content of the unenriched and enriched CW-DHP samples were similar, indicating that the exogenous isotopes added to the culture medium did not inhibit the normal growth and development of ginkgo cambial tissues.

#### 2.2.3. Solid-State CP/MAS ^13^C-NMR Analysis

The dehydrogenation polymer (DHP) formed by the polymerisation of the coniferyl alcohol monomer was called “conventional DHP”. Compared to natural lignin or milled-wood lignin (MWL), conventional DHP was characterised by significant amounts of β-β and β-5 structures, as well as end-groups which were mainly of the coniferyl alcohol type, while the β-O-4 linkages were present to a lesser extent [30,31,32]. The CW-DHP prepared by simulating natural lignification conditions contains complete wood cell walls, and its molecular weight was hundreds of times larger than that of conventional DHP. In CW-DHP, the frequency of β-O-4 substructures was higher than that of conventional DHP, which was closer to native lignin. The combined frequency of β-5, β-β, and β-1 and the frequency of the coniferyl alcohol/coniferaldehyde side chain were lower than that of conventional DHP and slightly higher than that estimated for ginkgo lignin [27,31,33]. Therefore, CW-DHP was more similar to natural lignin than conventional DHP.

Stable isotope enrichment and subsequent solid or liquid NMR analysis have previously been applied to wood xylem [23,24,25,28,34]. To distinguish the isotope-labelled signals of the carbon atoms found in the lignin side chain from those found in non-labelled samples, solid-state cross-polarization magic angle spinning (CP/MAS) ^13^C-NMR differential spectroscopy was used. In the difference spectra, all carbon signals, except the ^13^C enhanced signal, are eliminated. Thus, peak areas in the difference spectra represent the frequency of enriched α-^13^C and are assigned to different types of α-carbon according to chemical shifts.

**Figure 2:**
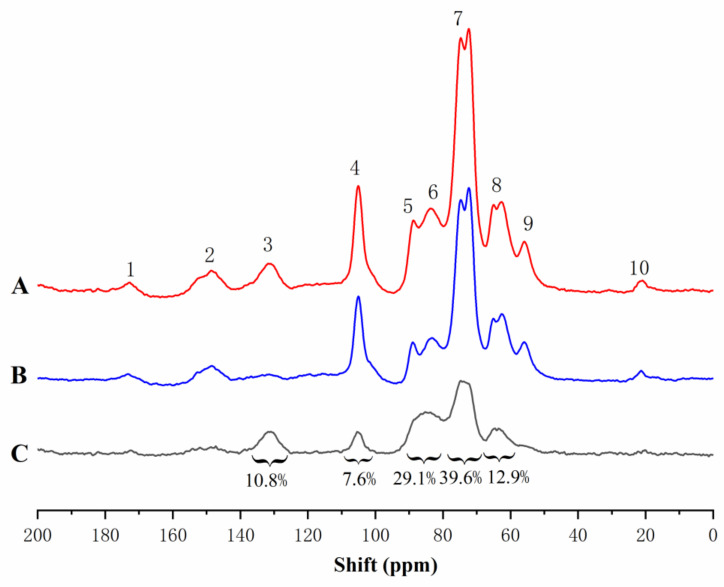
Solid-state CP/MAS ^13^C-NMR spectra of Ginkgo CW-DHP: (**A**) ^13^C-^2^H-enriched ginkgo CW-DHP; (**B**) unenriched ginkgo CW-DHP; and (**C**) difference spectrum obtained by subtracting spectra (**B**) from spectra (**A**).

The solid-state CP/MAS ^13^C-NMR spectra of enriched and unenriched ginkgo CW-DHP and their difference spectrum are shown in Figure 2. The assignment of their signals is shown in Table 3. The difference spectrum showed five broad peaks. From the integrated area in the difference spectrum, 80.1–67.9 ppm accounted for 39.6% of the total area, which mainly consisted of β-O-4 substructures [11,22,35]; the area at 93.1–80.7 ppm was 29.1%, which was mainly β-5, β-β, and Cα-O-R (R was glycosyl) in lignin [26], and the area at 67.9–58.0 ppm which accounted for 12.9%, and which mainly could be assigned to the β-1 substructures in lignin. The above results confirmed that the lignin produced in ginkgo CW-DHP had the structural characteristics of typical DHP. The area at 100.5–110.2 ppm accounted for 7.6%, demonstrating that ginkgo CW-DHP had a limited amount of ketal linkages formed between carbohydrates and lignin C-α [26,36]. The area at 140.5–124.7 ppm was 10.8%, and this region was principally CαH = CH in coniferyl alcohol [37], indicating a higher amount of coniferyl alcohol than protolignin in the CW-DHP.

#### 2.2.4. XRD Characterisation of CW-DHP and the Fractions

**Figure 3:**
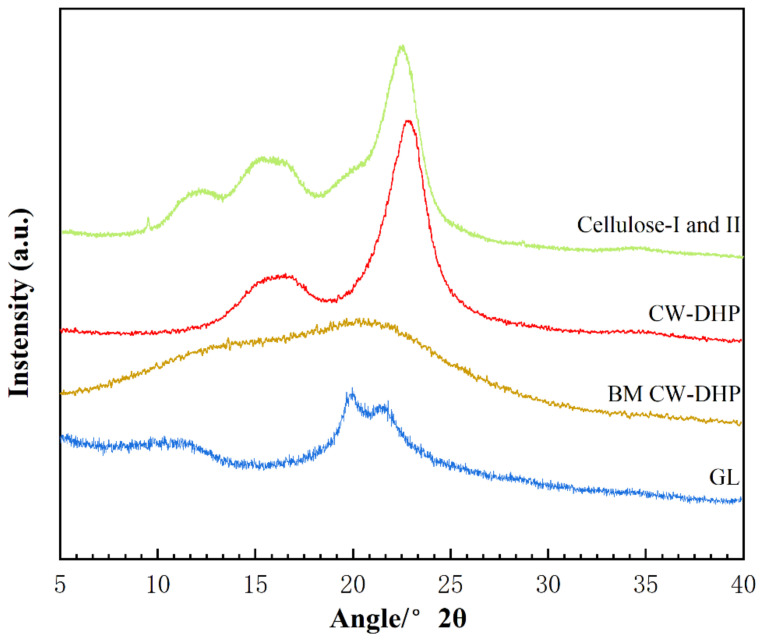
X-ray diffraction spectra of CW-DHP, BM CW-DHP, GL enriched with [^13^C-^2^H] and Cellulose I and II.

The X-ray diffraction (XRD) spectra observed of CW-DHP, ball-milled (BM) CW-DHP, glucan–lignin complex (GL) labelled with dual isotope, and cellulose I and II were shown in Figure 3. The characteristic peaks of CW-DHP were located at 2θ = 15°, 16.5° and 22.5°, respectively, corresponding to the (1 −1 0), (1 −1 0), and (1 −1 0) crystal planes of cellulose I [38,39]. After 12 h of complete ball milling, the CW-DHP was physically altered, with the majority of the crystalline structure in the cellulose being destroyed [18]. The CW-DHP after ball milling was completely dissolved in the DMSO/TBAH system, and the crystalline morphology of the cellulose I of the original CW-DHP was converted into cellulose II in the precipitated fraction GL, suggesting that cellulose is the major glucan component in LCC. The new characteristic peaks of cellulose II appeared in the XRD spectra of GL at 2θ = 12.5°, 20° and 22.3°, corresponding to (1 −1 0), (1 1 0) and (0 2 0), respectively [40,41].

#### 2.2.5. Molecular Weight Evaluation of LCC Fractions

The molecular weights and polydispersity results of different LCC fractions are shown in Table 4. The molecular weights’ order was acetylated GL (Ac-GL) > acetylated glucomannan–lignin complex (Ac-GML) > xylan–lignin complex (XL). These results are in agreement with the common knowledge that the order of the molecular size of the carbohydrates is cellulose > glucomannan > xylan. Due to its high molecular weight, the GL fraction was precipitated promptly after dispersion in water. The hemicelluloses in softwood mainly consist of glucomannan. Barium ions readily form insoluble complexes with mannans through interactions between the ions and the vicinal *cis-hydroxyl* groups on carbons 2 and 3 of the mannose units. Other polysaccharides, e.g., xylan, are not precipitated because they have no such *cis-hydroxyl* group structure [42]. The polydispersity of Ac-GL was the least, which indicated that the molecular weights of Ac-GL were generally too large. After enzymatic hydrolysis, the molecular weights of Ac-En-GL and En-XL were greatly reduced, part of the polysaccharides in the complexes were effectively removed, and the distribution of molecular weights became wider (Mw/Mn > 1.6).

### 2.3. Characterisation of the Glucan–Lignin Complex

#### 2.3.1. ^13^C-NMR Analysis of the Glucan–Lignin Complex (GL)

**Figure 4:**
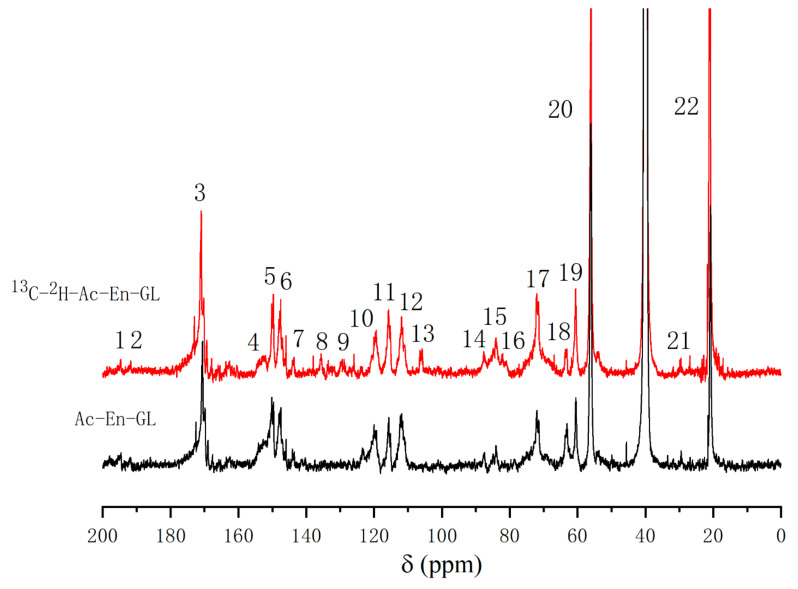
^13^C-NMR spectra of Ac-En-GL and Ac-En-GL-[^13^C-^2^H] fractions from ginkgo CW-DHP.

After enzymatic hydrolysis of GL via cellulase and hemicellulase, many of the β-1, 4-glycosidic bonds between the glucan units were cleaved, resulting in more explicit chemical structure information between lignin and glucan. After enzymatic hydrolysis, the major components are lignin and glucan chemically bonded to lignin. To obtain Ac-En-GL, the GL after enzymatic hydrolysis (En-GL) was acetylated to improve solubility. The ^13^C-NMR spectra of the Ac-En-GL and [^13^C-^2^H]-Ac-En-GL are shown in Figure 4. The assignments of important signals are listed in Table 5. The signal of the methoxy group at 56.4 ppm (No. 20) was used as a reference to compare the other signal shifts. In the 110–150 ppm aromatic region, there was no obvious difference between the spectra of Ac-En-GL and ^13^C-enriched Ac-En-GL. However, in the aliphatic region (50–110 ppm and 191–194 ppm), some signals were greatly enhanced due to the ^13^C enrichment of lignin side chains. This indicated that exogenously supplied coniferin-[α-^13^C] participates in lignin metabolism without interfering with the normal lignin biosynthesis.

Comparing the spectrum of Ac-En-GL-[^13^C-^2^H] with that of Ac-En-GL, 194.4 ppm (No. 1) and 191.0 ppm (No. 2), signal enhancements could be assigned to the α-CO and vanillin α-CHO, respectively, and the enhanced signal at 153.0 ppm (No. 4) could be assigned to cinnamaldehyde C-α. An enhanced signal that appeared at 143.7 ppm (No. 7) could be assigned to cinnamic acid C-α. An enhanced signal at 129.7 ppm (No. 9) was related to the -CαH = CH- in the guaiacyl side chain. The signal at 105.8 ppm (No. 13) was enhanced by ^13^C enrichment; according to the ^13^C-NMR spectra of the model compound, the signal was considered to be the ketal linkages between C-α of the lignin side chain and glucan, the signal could also be assigned to C-1 in glucose [11,43]. The enhanced signals at 87.4 ppm (No. 14) and 84.0 ppm (No. 15) were assigned to C-α in phenylcoumaran and pinoresinol substructure. The signal at 82.0 ppm (No. 16) was obvious in ^13^C enriched Ac-En-GL, which was considered as C-α with benzyl ether linkages to glucan [11,26]. The signal at 72.1 ppm (No. 17) was significantly enhanced, and could be assigned to C-2 in glucose, C-α in β-aryl ether substructure and C-6 in glucan with ether linkage to lignin [29]. The (No. 18) and (No. 19) enhanced signal peaks near 70–60 ppm corresponded to C-γ in β-5, C-γ in β-arylether and C-6 in glucose, respectively.

#### 2.3.2. ^1^H-NMR Analysis of the GL

**Figure 5:**
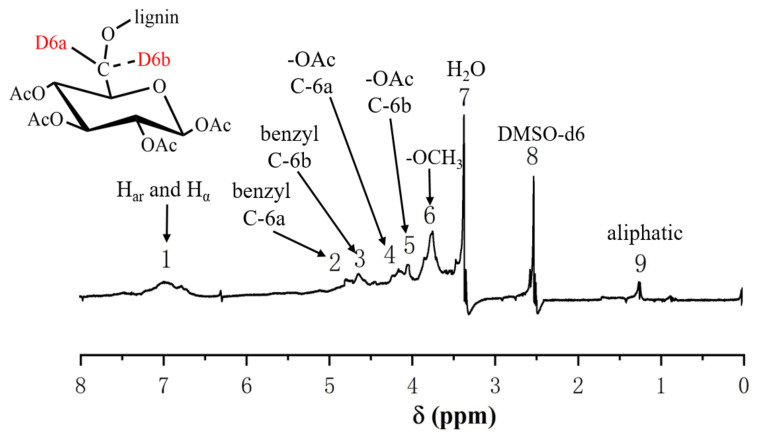
^1^H-NMR differential spectrum of Ac-En-GL obtained from ginkgo CW-DHP by subtracting the spectrum of Ac- En-GL-[^13^C-^2^H] from the spectrum of Ac-En-GL.

To further investigate the chemical bonds between the lignin side chain and glucan C-6, the acetyl group (δ = 1.9–2.4 ppm) was used as a reference to compare the other signal shifts. The ^1^H-NMR differential spectrum of Ac-En-GL (Figure 5) was obtained by subtracting the ^1^H-NMR spectrum of the experimental group from the ^1^H-NMR spectrum of the control group. According to the ^1^H-NMR analysis of processed hexose [44], a pair of shoulder-shaped signal peaks at 4.68 ppm (No. 2) and 4.59 ppm (No. 3) was identified in benzyl ether with glucan C-6 and designated as D-6a and D-6b, indicating benzyl ether linkages between glucan C-6 and lignin [26,45]. The resonance signal at 4.15 ppm (No. 4) and 4.03 ppm (No. 5) of D-6a and D-6b could be assigned to the esterified glucan because of the acetylation of En-GL [46,47]. The above analysis found benzyl ether linkages between glucan C-6 and lignin side chain C-α in ginkgo CW-DHP.

### 2.4. Characterisation of Glucomannan—Lignin Complex and Xylan—Lignin Complex

#### 2.4.1. ^13^C-NMR Analysis of the Glucomannan—Lignin Complex (GML)

**Figure 6:**
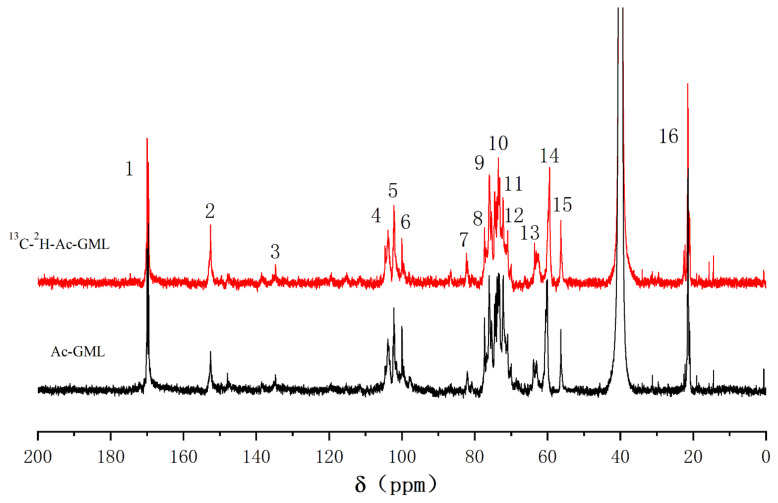
^13^C-NMR spectra of Ac-GML from ginkgo CW-DHP.

To enhance the solubility in DMSO-*d*_6_, the GML fraction was pre-acetylated to obtain Ac-GML. The ^13^C-NMR spectra of the Ac-GML and [^13^C-^2^H]-Ac-GML are shown in Figure 6. The signal of the methoxy group at 56.3 ppm (No. 15) was used as a reference. The signal peaks at 152.5 ppm (No. 2) and 63.5 ppm (No. 13) were significantly enhanced and assigned to C-α of cinnamaldehyde and C-α of β-1 substructures, respectively [20,48]. The signal at 103.8 ppm (No. 4) could be assigned to C-1 in glucose and C-1 in mannose. The signal at 82.2 ppm (No.7) was enhanced by ^13^C enrichment, which was characteristic of the benzyl ether bonds between C-α of the lignin side chain with the glucomannan [11,49,50].

#### 2.4.2. ^1^H-NMR Analysis of the GML

**Figure 7:**
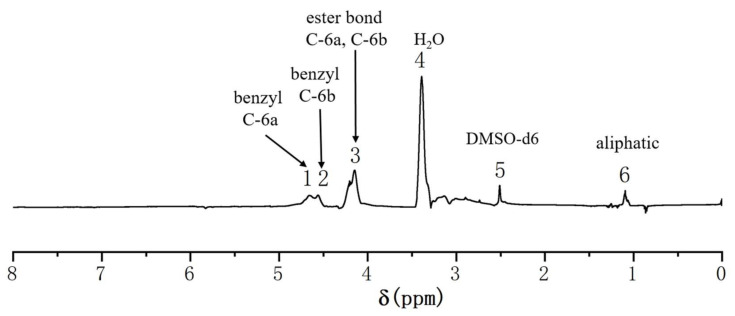
^1^H-NMR differential spectrum of Ac-GML obtained from ginkgo CW-DHP by subtracting the spectrum of Ac-GML-[^13^C-^2^H] from the spectrum of Ac-GML.

In the ^1^H-NMR differential spectrum (Figure 7), the acetyl group (δ = 1.9–2.4 ppm) was selected as a reference. The pair of shoulder signals at 4.66 ppm (No. 1) and 4.56 ppm (No. 2) was assigned to the benzyl ether linkages generated by glucomannan C-6 and lignin [20,44,45]. There were ester bonds at the non-etherified glucomannan C-6 after the acetylation of GML, and the enhanced signal of the ester linkages at 4.15 ppm (No. 3) arising from the acetyl group in lignin and glucomannan. Comprehensive ^13^C-NMR spectrum analysis of Ac-GML showed benzyl ether linkages between the lignin side chain C-α and glucomannan C-6.

#### 2.4.3. ^13^C-NMR Analysis of the Xylan—Lignin Complex

**Figure 8:**
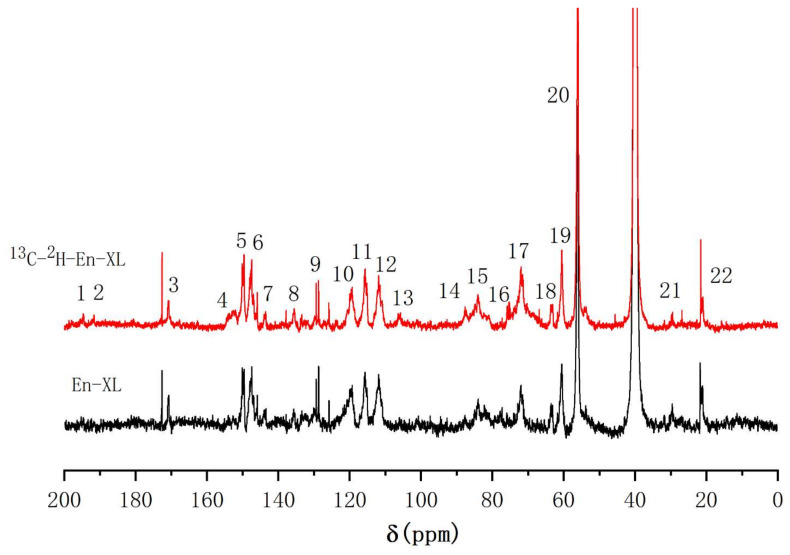
^13^C-NMR spectra of En-XL from ginkgo CW-DHP.

To reduce the signal from xylan components without linkage to lignin, the XL was hydrolysed with xylanase to obtain enzymatic hydrolysis XL (En-XL). As the En-XL has better solubility, acetylation was not required. The ^13^C-NMR spectra of En-XL and [^13^C-^2^H]-En-XL are shown in Figure 8 with the methoxy group at 56.4 ppm (No. 20) used as a reference. The signal enhancements at 194.4 ppm (No. 1) and 191.0 ppm (No. 2) in the lignin side chain could be assigned to α-CO and vanillin α-CHO, respectively, and the enhanced signal at 153.0 ppm (No. 4) could be assigned to cinnamaldehyde C-α. The signal at 105.5 ppm (No. 13) was enhanced by ^13^C enrichment and was considered to be the ketal linkages formed between the C-α of the lignin and xylan [11,26]. The enhanced signals at 87.4 ppm (No. 14) and 84.0 ppm (No. 15) were assigned to C-α in phenylcoumaran and pinoresinol substructure, erspectively, and the signal at 73.7.1 ppm (No. 16) could be assigned to C-2 and C-3 in xylan [11]. The signal at 72.1 ppm (No. 17) was significantly enhanced and could be assigned to C-α in β-aryl ether substructure [51]. The signal at 63.2 ppm (No.18) was characteristic of the C-γ in β-5, C-α in spirodienone and C-5 in xylan [52].

Xylan is biosynthesised in plants by converting D-glucose to glucuronic acid, which is subsequently converted into xylan through a series of reactions [53,54]. In this process, the ^2^H at the C6 position of D-glucose-[6-^2^H_2_] was hydrolysed and eliminated, therefore the ^1^H-NMR of the En-XL-[^13^C-^2^H] was not studied.

### 2.5. Chemical Structures

**Figure 9:**
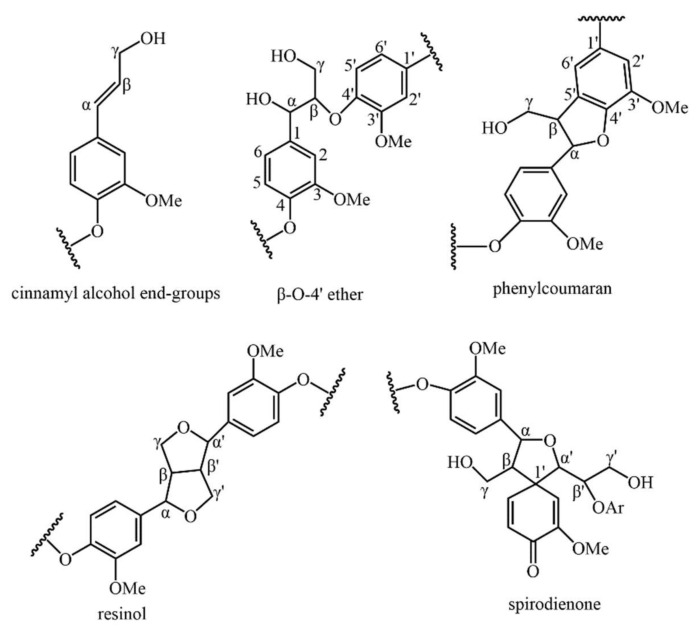
Major linkages between lignin substructural units in Ginkgo CW-DHP.

**Figure 10:**
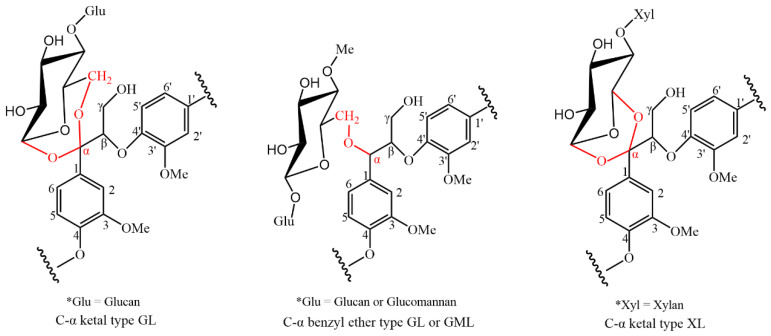
LCC linkages in ginkgo CW-DHP.

The related lignin substructures and the LCC linkages structure in the ginkgo CW-DHP are shown in Figure 9 and Figure 10.

## 3. Materials and Methods

### 3.1. Materials

Five-year-old *Ginkgo biloba* L. trees were obtained from the Wuhan Botanical Garden (Wuhan, China). Sodium acetate-1-^13^C, D-glucose-[6-^2^H_2_], hemicellulase (*Aspergillus niger*, ≥1500 unites/g), β-glucosidase (*almond*, ≥7000 unites/g) and dual antibiotics (*penicillin-streptomycin*) were purchased from Sigma-Aldrich (St. Louis, MO, USA). Cellulase (*Onozuka* RS, 16,000 unites/g) was purchased from Aladdin Reagent (Shanghai, China). Acetyl bromide, tetrabutylammonium hydroxide (TBAH, 40% *w*/*w* in water), *N*-methylimidazole carboxymethoxyamine hemihydrochloride (AOA) and xylanase (*Aspergillus oryzae*, ≥2500 unites/g) were purchased from Macklin Reagent (Shanghai, China). All other chemicals are of analytical grade.

### 3.2. Preparation of Ginkgo CW-DHP and Lignin–Carbohydrate Complex Fractions

#### 3.2.1. Synthesis of Coniferin

**Figure 11:**
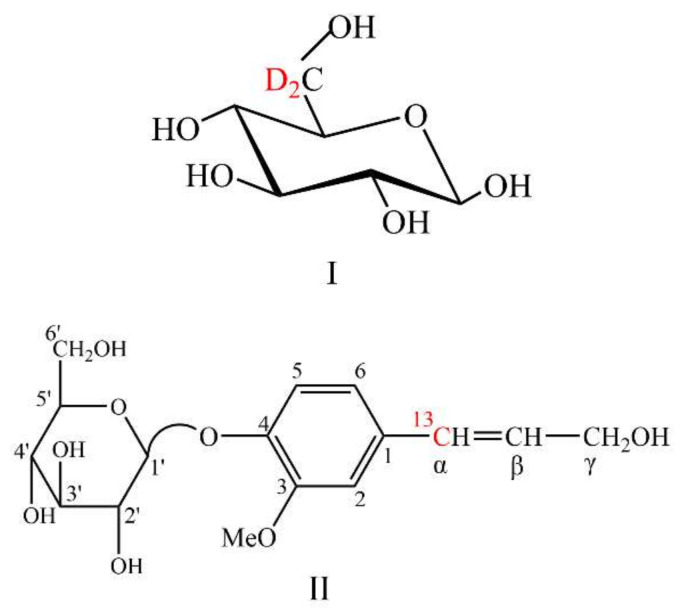
Chemical structures of D-glucose-[6-^2^H_2_] (**I**) and coniferin-[α-^13^C] (**II**).

**Figure 12:**
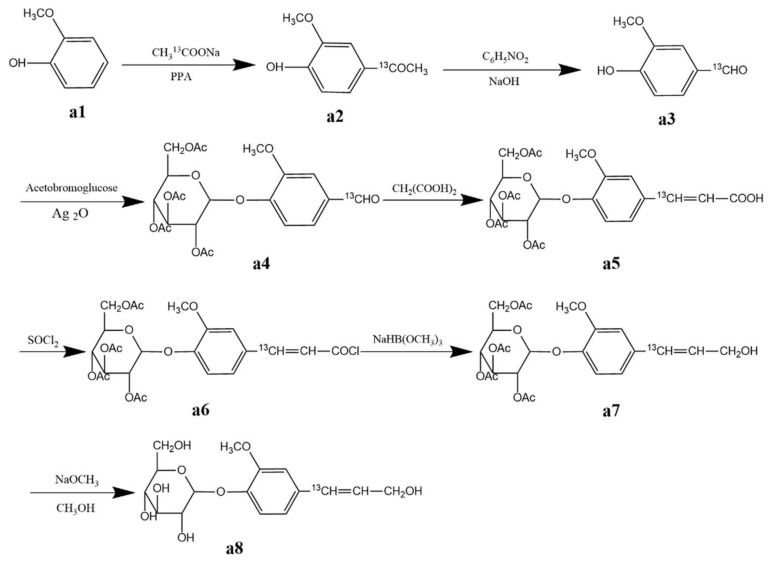
Synthesis of coniferin-[α-^13^C].

Coniferin, both unenriched and ^13^C-enriched at the side chain C-α, was synthesised using the methods described in [25,55,56]. The chemical structures of the isotopically enriched compounds are shown in Figure 11. The Synthesis of coniferin-[α-^13^C] is shown in Figure 12.

The yield and melting point of the product obtained in each synthetic step are as follows:

a2: 4-Acetylguaiacol-[α-^13^C]. Yield: 55.4%, Mp: 113–115 °C.

a3: Vanillin-[α-^13^C]. Yield: 80.3%, Mp: 80–81 °C.

a4: Vanillin (2,3,4,6-tetra-O-acetyl)-β-D-glucoside-[α-^13^C]. Yield: 77.6%, Mp: 142.5–144.5 °C.

a5: Ferulic acid (2,3,4,6-tetra-O-acetyl)-β-D-glucoside-[α-^13^C]. Yield: 98.6%, Mp: 206–208 °C.

a7: Coniferyl alcohol (2,3,4,6-tetra-O-acetyl)-β-D-glucoside-[α-^13^C]. Yield: 74.8%, Mp: 143–146 °C.

a8: Coniferin-[α-^13^C]. Yield: 79.5%, Mp: 184–185 °C.

#### 3.2.2. Preparation of Gingko CW-DHP

A growing ginkgo tree (approximately 20–30 cm in diameter at breast height [DBH]) was cut down in early June; its bark was removed, and its soft cambial tissue was collected and stored in a deep freezer at −80 °C.

**Figure 13:**
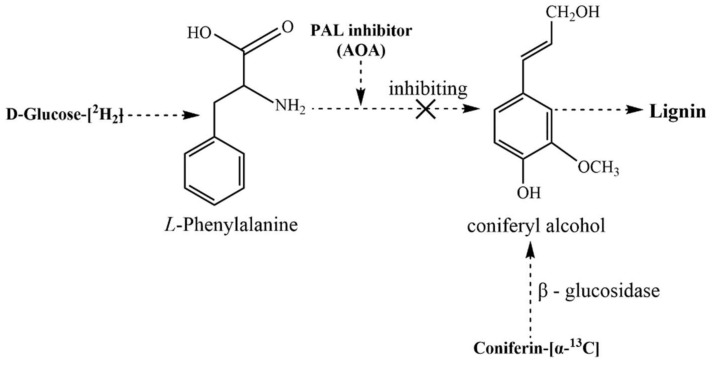
Inhibiting of D-glucose-[6-^2^H_2_] transformation to lignin and metabolism of coniferin-[α-^13^C].

In a sterile environment at 25 °C, 200 μL dual antibiotics were added to 80 mL phosphate buffer (0.2 M, pH 6.0). Following that, 500 mg of α-^13^C-enriched or unenriched coniferin, 600 mg of [6-^2^H_2_]-enriched or unenriched D-glucose and *N*-methylimidazole carboxymethoxyamine hemihydrochloride (AOA) were dissolved in the buffer. Thirty grams of ginkgo cambial tissues were added to the mixture, then it was placed into a sterile incubator and the incubator was opened for air exchange every 12 h. The AOA are inhibitors of phenylalanine ammonia-lyase (PAL) [26,57]; the conversion of D-glucose-[6-^2^H_2_] to lignin could be inhibited by the addition of AOA. Coniferin-[α-^13^C] can be converted into lignin under the action of β-glucosidase in the cell. This mechanism is shown in Figure 13. Ten days later, an additional 100 mg of α-^13^C-enriched or unenriched coniferin, 300 mg of [6-^2^H_2_]-enriched or unenriched D-glucose, 42 U β-glucosidase and 200 μL dual antibiotics were added.

The culture was stopped after 18 days. The samples were washed three times successively with 8 M urea, four times with distilled water and three times with methanol and diethyl ether (100 mL) to remove residual coniferin and enzymes, and then they were vacuum dried to obtain ginkgo CW-DHP.

#### 3.2.3. Preparation of Lignin–Carbohydrate Complex Fractions

**Figure 14:**
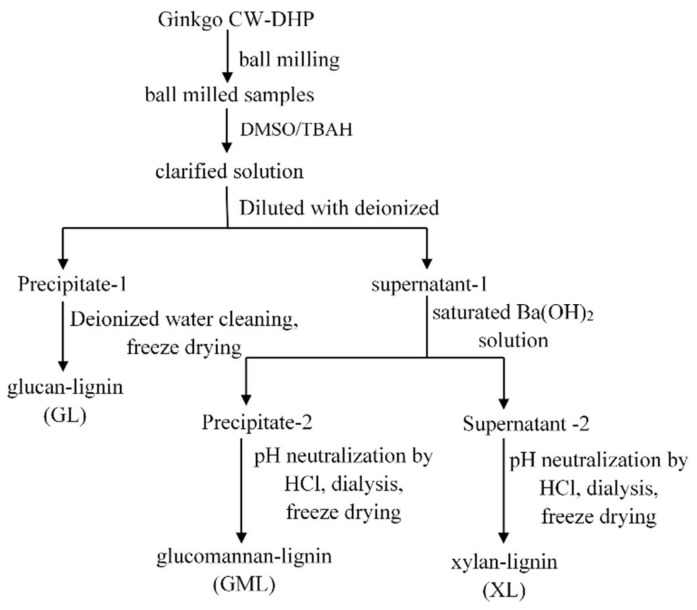
Fractionation of LCC from Ginkgo CW-DHP.

As shown in Figure 14, ginkgo CW-DHP was ground into 40–60 mesh. After complete drying, the samples were further ground in a water-cooled vibrating ball mill for 12 h to yield ball-milled samples, which were then completely dissolved in an ionic liquid system composed of DMSO (5 mL) and TBAH (5 mL). The solution was then poured into deionised water (100 mL) and stirred to obtain two phases: precipitate-1 and supernatant-1. Precipitate-1 was continuously washed with deionised water until it reached pH 7 and then was freeze-dried to obtain enriched GL. After adding saturated Ba(OH)_2_ solution (100 mL) into supernatant-1, precipitate-2 and supernatant-2 were separated by centrifugation. As a result, enriched GML can be recovered by neutralising precipitate-2 with HCl solution, followed by dialysis (the molecular weight cut-off is 1000 Da) and freeze-drying. Similarly, enriched XL can be obtained from supernatant-2 by neutralizing the solution with HCl, followed by dialysis and freeze-drying.

#### 3.2.4. Enzymatic Hydrolysis and Acetylation of LCC Fractions

In 50 mL of 0.1 M acetic acid/sodium acetate buffer (pH 4.8), 8000 U cellulase and 300 U hemicellulase were dissolved. The filtrate was collected and stored at 4 °C. Under continuous shaking at 50 °C, 200 mg GL was hydrolysed with 5 mL of the above enzyme solution and 8 mL of acetic acid/sodium acetate buffer, with two drops of toluene added as a preservative. After 48 h of enzymatic hydrolysis, the residue was hydrolysed again with the above method. The precipitate was rinsed with deionized water after centrifugation, then freeze-dried to yield enzymatic hydrolysis GL (En-GL).

1250 U xylanase was dissolved in 50 mL of 0.1 M acetic acid/sodium acetate buffer (pH 4.8). The filtrate was collected and stored at 4 °C. According to the En-GL, as mentioned above in the enzymatic hydrolysis method, 200 mg XL was processed to obtain enzymatic hydrolysis XL (En-XL).

For 2 h, 100 mg of GL, En-GL or GML was dissolved in DMSO/*N*-methylimidazole (2:1, *v*/*v*). The mixture was stirred for 1.5 h after adding 0.5 mL acetic anhydride and precipitated in water to complete acetylation. The acetylated sample was obtained by centrifugation and freeze-drying. Parallel tests were carried out for samples with and without isotope labeling using the same parameters. It was ensured that influencing factors other than the samples were consistent, so that each group of samples could reach the same degree of acetylation.

### 3.3. Characterisation of Gingko CW-DHP

#### 3.3.1. Determination of ^13^C and ^2^H Abundance

The C isotope value δ^13^C (Vienna Pee Dee Belemnite [VPDB]) and the H isotope value δD (Vienna Standard Mean Ocean Water [VSMOW]) of 1 mg of gingko CW-DHP in the experimental and control groups were evaluated via an elemental analyser (FLASH2000, Thermo Fisher Scientific GmbH, Dreieich, Germany) combined with an isotope ratio mass spectrometer (Delta V, Thermo Fisher Scientific GmbH, Dreieich, Germany). The samples’ ^13^C/^12^C and ^13^Cα/^12^Cα values were calculated using Equations (1) and (2)

^13^C/^12^C = (1 + δ^13^C ÷ 1000) × 1.105765% (1)

^13^Cα/^12^Cα = (^13^C/^12^C − 1.0762%) ÷ 0.1989 × 10 + 1.0762% (2)

where ^13^C/^12^C is the ratio of ^13^C to ^12^C in the sample, δ^13^C (‰) is the C isotope value of the sample, 1.105765% is the ^13^C isotope abundance of the standard VPDB, ^13^Cα/^12^Cα is the ^13^C and ^12^C isotopic ratios of Cα in the lignin structural units of the sample, 1.0762% is the ^13^C isotope values in intact ginkgo wood, 0.1989(%) is the lignin contents in ginkgo CW-DHP and 10 is the ratio of total carbons of guaiacyl propane moiety to the number of C-α to in the lignin.

The samples’ D/H and D6/H6 values were calculated using Equations (3) and (4)

D/H = (1+ δD ÷ 1000) × 0.015575%(3)

D6/H6 = (D/H − 0.013505%) ÷ 0.45 × 5 + 0.013505%(4)

where D/H is the ratio of D to H in the sample, δD (‰) is the H isotope value of the sample, 0.015575% is the D isotope abundance of the standard VSMOW, D6/H6 is the isotope ratio of D to H in cellulose at position 6 in the sample, 0.013505% is the D isotopic value in intact ginkgo plants, 0.45(%) is the cellulose contents in ginkgo CW-DHP, and 5 is the ratio of the number of total hydrogens in a glucose unit in cellulose to H6 in the cellulose structures.

#### 3.3.2. Determination of the Lignification of Ginkgo CW-DHP

The lignin contents of the samples were determined by the improved acetyl bromide method [58] to be λ = 280 nm and the sum of acid-insoluble and acid-soluble lignin. An absorptivity value of 18.8 L/g^−1^ cm^−1^ (at 280 nm, acetyl bromide method) was used. In this experiment, ginkgo wood mill was used as the standard sample and lignin values in the ginkgo CW-DHP were calculated using Equation (5)

X = (K × A)/B × 100%(5)

where B is the absorbance of the standard ginkgo wood mill, K is the total lignin contents of the standard ginkgo wood mill, and A is the absorbance of the CW-DHP.

#### 3.3.3. Determination of Solid-State ^13^C-NMR Spectroscopy

The solid-state CP/MAS ^13^C-NMR was recorded on an Avance III 600-MHz solid-state NMR spectrometer with a solid probe (Bruker, Billerica, MA, USA). The experimental conditions were as follows: a temperature of 14.1 °C, 3 ms contact time, 0.05 s reception time, the magic angle spinning frequency fixed at 10 kHz, a pulse width of 35 kHz and a pulse delay of 2 s. Each sample was accumulated approximately 5000 times. All of the spinning side bands are very small, by fixing the spinning frequency, the small spinning side bands will not contribute to the difference spectra [59].

#### 3.3.4. Determination of XRD

The X-ray Diffraction system (Empyrean Sharp, Panaco, The Netherlands) was used to analyse the crystallisation characteristics of fully dried samples with a CuKα radiation wavelength of 0.154 nm. Diffractograms were achieved by scanning from 5° to 40° (2θ) at a rate of 5°/min, a step size of 0.02° and a divergence slit width of 1°.

#### 3.3.5. Determination of Molecular Weights

Gel permeation chromatography (GPC) was determined by a Shimadzu LC-20AD HPLC system (Shimadzu, Kyoto, Japan) equipped with a shim pack GPC-803D column (Shimadzu, Kyoto, Japan) and a refractive index (RI) detector (Shimadzu, Kyoto, Japan) at a flow rate of 1 mL/min at 40 °C with *N, N*-dimethylformamide (DMF) as eluent. The sample (2 mg) was dissolved in 1 mL DMF, and 25 μL of this solution was injected into GPC. Calibration curves were established based on four monodisperse polystyrene standards with molecular weights ranging from 2900 to 19,800 g/mol. The data were analysed using liquid chromatography solution software (Shimadzu, Kyoto, Japan).

#### 3.3.6. Determination of ^13^C-NMR and ^1^H-NMR Spectroscopy

First, 80 mg of the samples was dissolved in 0.5 mL of DMSO-*d*6. Then, all NMR spectra were recorded on a Bruker Avance III 500-MHz spectrometer (Fällanden, Switzerland) equipped with a φ5 mm broad-band fluorine observation (BBFO) probe at a temperature of 25 °C. The ^13^C NMR spectra were recorded in the FT mode at 100.6 MHz with the following conditions: 1.75-s pulse delay and 0.94-s reception time, with the data points collected at 32 kbit and after accumulating 20,000 scans. The ^1^H-NMR spectrum was recorded with the following conditions: 4.3-s pulse delay and 0.74-s acquisition time, with the data points collected at 32 kbit and after the accumulation of 500 scans.

## 4. Conclusions

1. Detection of the ^13^C and ^2^H abundance of ginkgo CW-DHP showed that exogenous coniferin-[α-^13^C] and D-glucose-[6-^2^H_2_] were involved in the normal metabolism of the soft ginkgo cambial tissues. Therefore, the lignin and cell wall polysaccharides were successfully labelled by ^13^C and D, respectively. Furthermore, the degree of lignification of ginkgo CW-DHP was increased by biological culture.

2. Solid-state CP/MAS ^13^C-NMR analysis revealed that the main lignin linkages include β-O-4, β-5, β-1 and β-β substructures, of which β-O-4 was the most abundant. In addition, ketal and ether linkages were formed between the C-α of lignin side chains and carbohydrates in ginkgo CW-DHP.

3. XRD examination indicated that the cellulose I of GL was converted into cellulose II during the separation process. The molecular weight was in the order of Ac-GL > Ac-GML > XL. After enzymatic hydrolysis, the molecular weight was significantly reduced, indicating the removal of carbohydrates without chemical bonds to the lignin macromolecule.

4. There were Cα-ketal linkages and Cα-benzyl ether linkages between glucan and lignin, while the lignin was linked with the glucomannan through Cα-benzyl ether linkages. Xylan and lignin were linked mainly by Cα-ketal linkages.

## Figures and Tables

**Table 1:** ^13^C and D abundance of Ginkgo CW-DHP.

Sample	^13^C(VPDB)	^13^Cα/^12^Cα (%)	D(VSMOW)	D6/H6 (%)
A	−27.18 (±2.21)	1.08	−132.90 (±4.20)	0.01
B	81.69 (±5.46)	7.10	1902.75 (±30.25)	0.37

Note: A: unenriched ginkgo CW-DHP; B: ^13^C-^2^H-enriched ginkgo CW-DHP.

**Table 2:** Comparison of lignin contents before and after the culture of the soft cambial tissues of a ginkgo tree.

Samples	Lignin Contents		
	Cambial Tissues	CW-DHP	Increase
unenriched sample	14.47% (±0.12%)	19.74% (±0.16%)	5.42%
^13^C-^2^H-enriched sample	14.47% (±0.12%)	19.89% (±0.14%)	5.27%

**Table 3:** Assignment of signals of CP/MAS ^13^C-NMR difference spectrum of Ginkgo CW-DHP.

Shift (δ, ppm)	Assignments	Ratio of Integrated Area
140.5–124.7	-CαH = CH- of coniferyl alcohol	10.8%
100.5–110.2	ketal linkages in carbohydrates and lignin C-α	7.6%
93.1–80.7	β-5, β-β and Cα-O-R (R was glycosyl)	29.1%
80.1–67.9	Cα in β-O-4	39.6%
67.9–58.0	Cα in β-1	12.9%

**Table 4:** Determination of molecular weights of different LCC fractions.

Samples	Mw (g/mol)	Mn (g/mol)	Mw/Mn
Ac-GL	3.9 × 10^5^	3.2 × 10^5^	1.22
Ac-En-GL	1.7 × 10^4^	6.4 × 10^3^	2.62
Ac-GML	2.7 × 10^4^	1.5 × 10^4^	1.80
XL	9.9 × 10^3^	6.3 × 10^3^	1.57
En-XL	5.3 × 10^3^	3.2 × 10^3^	1.65

**Table 5:** Assignments of ^13^C-NMR spectra of Ac-En-GL from ginkgo CW-DHP.

Signal	δ^13^C (ppm)		Assignments
	Ac-En-GL		
	^13^C-Enriched	Control	
1	194.6	194.3	α-CO, and α-CHO in vanillin
2	191.5	191.7	α-CHO
3	170.7	169.9	-C = O in acetyl group
4	153.0	153.2	C-4 in G, α-etherified; C-α in cinnamaldehyde
5	150.1	150.6	C-3 in G, α-etherified; C-4 in G, non-etherified
6	147.5	148.5	C-3 in G, non-etherified
7	143.7	143.3	C-α in cinnamic acid; C-4 in phenylcoumaran substructures
8	135.6	-	C-1 in G, non-etherified
9	129.7	-	C-1 in G with C-α in -CαH = CH- of coniferyl alcohols
10	119.5	119.3	C-6 in G, non-etherified
11	115.7	115.7	C-6 in β-5; C-5 in G, non-etherified
12	111.5	111.3	C-2 in G, non-etherified
13	105.8	-	C-α with ketal linkages; C-1 in glucose
14	87.4	87.2	C-α in phenylcoumaran
15	84.0	84.1	C-β in β-arylether; C-α in pinoresinol
16	82.5	-	C-α etherified to glucan
17	72.1	72.5	C-α in β-arylether; C-6 in glucan with ether linkage; C-2 in glucose
18	63.6	63.3	C-γ in phenylcoumaran
19	60.5	61.1	C-γ in β-arylether; C-6 in glucose
20	56.4	56.2	-OCH_3_
21	29.5	29.3	Unknown
22	21.3	21.9	-CH3 in acetyl group

## Data Availability

The data presented in this study are available in the manuscript.

## Sample Availability

Samples of the compounds are not available from the authors.
